# Local Competition and Metapopulation Processes Drive Long-Term Seagrass-Epiphyte Population Dynamics

**DOI:** 10.1371/journal.pone.0057072

**Published:** 2013-02-21

**Authors:** Delphine Lobelle, Emma J. Kenyon, Kevan J. Cook, James C. Bull

**Affiliations:** 1 School of Life Sciences, University of Warwick, Coventry, United Kingdom; 2 College of Medical and Dental Sciences, University of Birmingham, Birmingham, Alabama, United Kingdom; 3 Natural England, Truro, United Kingdom; Swansea University, United Kingdom

## Abstract

It is well known that ecological processes such as population regulation and natural enemy interactions potentially occur over a range of spatial scales, and there is a substantial body of literature developing theoretical understanding of the interplay between these processes. However, there are comparatively few studies quantifying the long-term effects of spatial scaling in natural ecosystems. A key challenge is that trophic complexity in real-world biological communities quickly obscures the signal from a focal process. Seagrass meadows provide an excellent opportunity in this respect: in many instances, seagrasses effectively form extensive natural monocultures, in which hypotheses about endogenous dynamics can be formulated and tested. We present amongst the longest unbroken, spatially explict time series of seagrass abundance published to date. Data include annual measures of shoot density, total above-ground abundance, and associated epiphyte cover from five *Zostera marina* meadows distributed around the Isles of Scilly, UK, from 1996 to 2011. We explore empirical patterns at the local and metapopulation scale using standard time series analysis and develop a simple population dynamic model, testing the hypothesis that both local and metapopulation scale feedback processes are important. We find little evidence of an interaction between scales in seagrass dynamics but that both scales contribute approximately equally to observed local epiphyte abundance. By quantifying the long-term dynamics of seagrass-epiphyte interactions we show how measures of density and extent are both important in establishing baseline information relevant to predicting responses to environmental change and developing management plans. We hope that this study complements existing mechanistic studies of physiology, genetics and productivity in seagrass, whilst highlighting the potential of seagrass as a model ecosystem. More generally, this study provides a rare opportunity to test some of the predictions of ecological theory in a natural ecosystem of global conservation and economic value.

## Introduction

The roles of spatial scale and structure in population dynamics remain a central theme in ecological research [1]–[5]. A classic approach to understanding the dynamics of spatially explicit populations is the metapopulation model of Levins [6], developed by Hanski [7] amongst others. Here, habitat with the potential to be colonized by a focal species is discretized, with the resulting patches classed as either occupied or vacant. This model gives rise to the familiar ‘blinking light’ dynamics, with local occurrence moving between the two states, as a result of colonization through dispersal, and extinction [7]. However, these dynamics rely on some strong, and often biologically unrealistic assumptions. Notably that potential habitat is infinite in extent and has no structure, with an equal probability of dispersal between neighbouring patches as those far apart; also, that local population dynamics are fast compared to metapopulation scale patch turnover, with local state moving between a stable equilibrium size or zero effectively instantly. As a result, there is broad consensus that in many systems it is necessary to understand population dynamics over a range of spatial and temporal scales in order to explain observed species distributions [2], [5], [8].

Despite these limitations, simple metapopulation models continue to be remarkably useful tools for understanding spatial dynamics in a wide variety of circumstances [9]–[12] One such area where the metapopulation scale approach has dominated our understanding is the study of epiphytes [13]–[15]. Epiphytes form an important component of many ecological communities, providing a substantial amount of additional biomass, carbon sequestration and niche diversity [16], [17]. However, much of what is known about epiphyte population dynamics is based on terrestrial forest systems [13], [14]. Here, epiphytes typically include lichens, bryophytes, ferns and relatively small flowering plants such as orchids and bromeliads. In these cases, it has often been assumed that distributions of these epiphytes are dependent on the presence or absence of host trees (metapopulation ‘patch-tracking’) rather than local environmental state variables (‘habitat-tracking’) [18], [19].

Where studies of forest epiphyte distributions do include local factors relating to habitat condition, these typically take the form of environmental gradients (such as microclimate and edge effects) rather than explicitly modelling feedback between host and epiphyte populations [20]–[23]. Whilst including independent variation in habitat structure adds considerably to a homogeneous metapopulation approach [19], [24], such models fail to capture important non-linearities inherent in many other, higher-turnover ecosystems. As a result, terrestrial forest communities may be unrepresentative of a range of widespread and valuable ecosystems where epiphytes are important; particularly in marine ecosystems such as those based on seagrasses or macrophytic algae.

To explore this, we turn to one of the most important [25] but still relatively poorly understood benthic ecosystems: that based on seagrass. Seagrasses are globally dispersed along coastlines, covering approximately 0.3 to 0.6 million km^2^ [26], [27]. Much of the value of seagrass meadows lies in their high levels of primary productivity, acting as a carbon and nutrient sink, providing a shelter for invertebrates or juveniles of fish species and protecting shorelines via wave attenuation and stabilization of sediments [25], [26], [28]. However, seagrasses are currently in rapid decline worldwide, due to a range of anthropogenic impacts, disease and climate change [29], [30]. As a result, there is considerable interest in understanding the drivers of seagrass population dynamics and a general appreciation that multiple spatial scales are important (for example, local density at the sub-metre scale [31]–[33], the influence of clonal expansion over tens of metres [8], [34], [35], or even metapopulation processes spanning oceans [36]).

Due to the substantial logistical and cost constraints inherent to the observation of a submerged natural ecosystem, many studies of seagrass dynamics take the form of laboratory or within season field trials, often focusing on single spatial scales of measurement [31], [32], [37]–[40] (although see [41], [42]). Therefore, there is a lack of fundamental knowledge about the long-term dynamics of seagrass and its associated flora and fauna, including a substantial epiphytic community that can account for over 30% of above ground seagrass meadow biomass [43], [44]. Typically, this epiphytic community is dominated by diatoms and rhodophytes (red algae) in a healthy seagrass community; with increasing occurrence of cyanobacteria, bryozoans, hydroids and brown, green or blue-green algae populations in sub-optimal environments [16], [44], [45].

**Figure 1:**
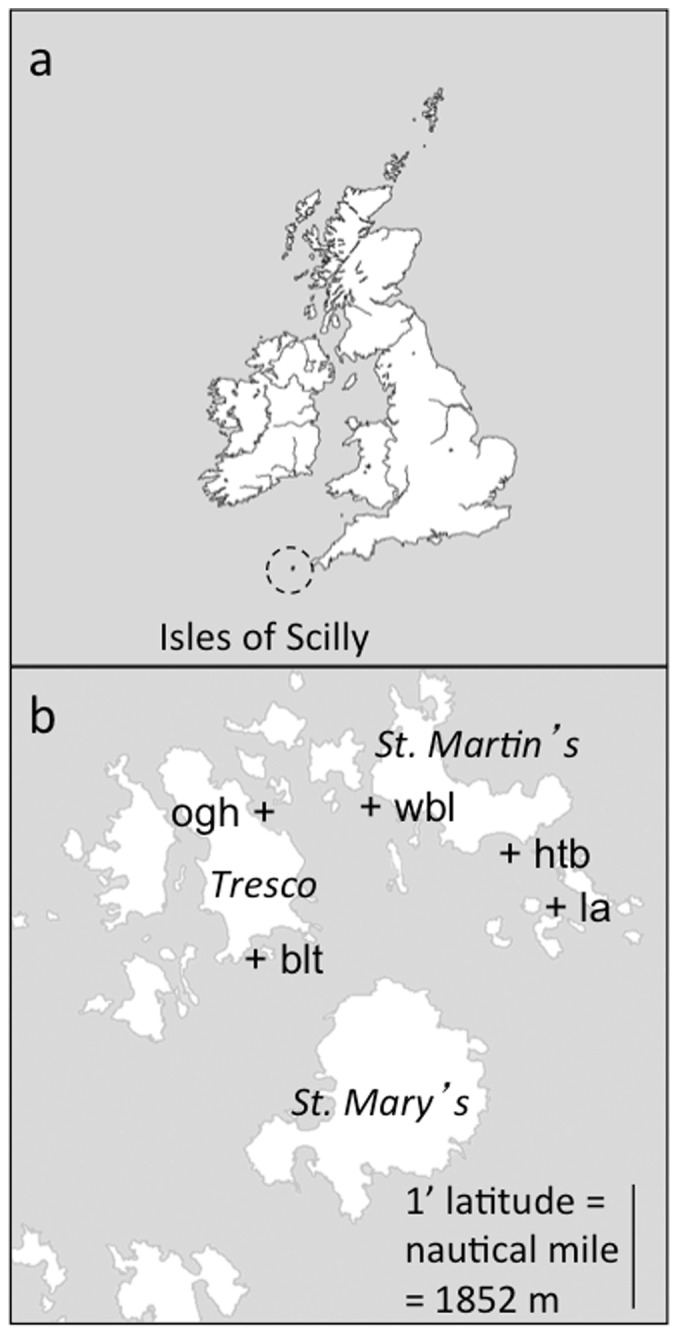
Study area location. Panel (a) the position of the Isles of Scilly relative to mainland United Kingdom; panel (b) the positions (marked **+**) of the five seagrass meadows sampled in this study: Broad Ledges Tresco (blt), Higher Town Bay (htb), Little Arthur (la), Old Grimsby Harbour (ogh), and West Broad Ledges (wbl).

In this study, we present novel data from an ongoing, spatially replicated study of a comparatively unimpacted temperate seagrass-epiphyte system. In this sub-tidal environment, there are no large grazing species, such as the geese that affect inter-tidal seagrass populations [46], [47], or the marine turtles and sirenians of tropical seagrass habitats [48], [49]. In addition, our choice of location is an archipelago with little industrial or agricultural impact or urbanization (Fig. 1). Here, seagrass grows substantially as a natural monoculture in which we are able to not only make rare baseline observations of a seagrass ecosystem not currently in overall decline, but also test theoretical predictions on ecological processes such as enemy-victim interactions [33] and competition, which would likely be masked by trophic complexities in many other natural environments.

Our aims were to test the hypotheses that: (1) epiphytes play an important role in the long-term dynamics of their seagrass hosts; and (2) inclusion of both local and metapopulation scale interactions are necessary to explain observed population dynamics. We developed a simple population dynamic model, fitting this to observed time series at different spatial scales using mixed-effects models and model averaging techniques. Whilst local scale effects dominate seagrass dynamics, we found approximately equal support for both local and metapopulation scale influences on epiphyte abundance.

## Results

### Empirical Patterns

**Figure 2:**
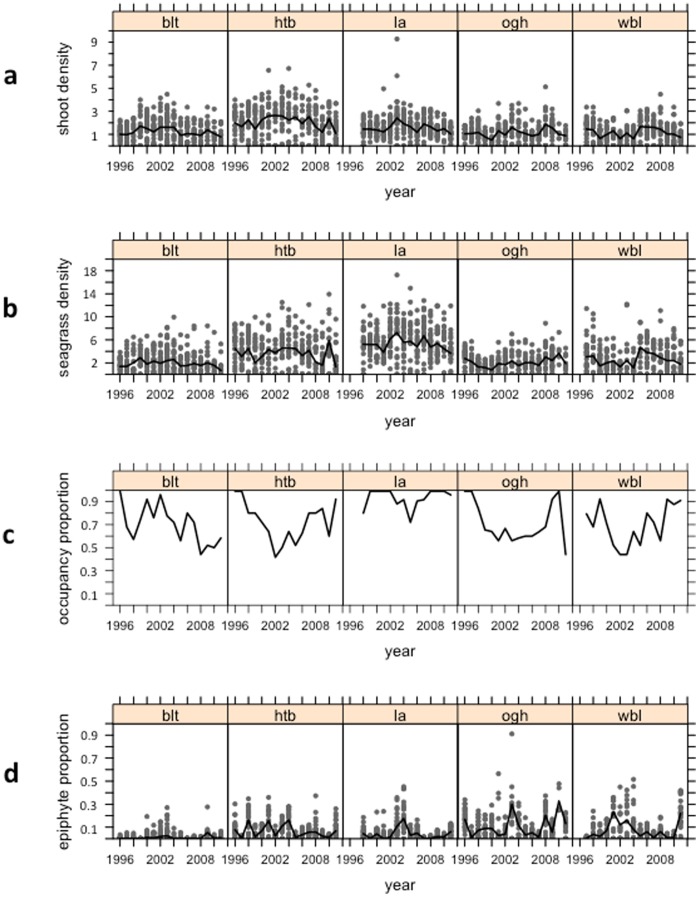
Time series of seagrass (Zostera marina) density and epiphyte cover. Panel (a) shoot density ×100 per square metre; panel (b) seagrass density ×100 per square metre (total metres of leaf per square metre of ground); panel (c) the proportion of quadrats occupied by seagrass in a given meadow; and panel (d) the average proportion of each leaf covered by epiphytes in a given quadrat. Grey dots show individual quadrat data; black lines show meadow averages for Broad Ledges Tresco (blt), Higher Town Bay (htb), Little Arthur (la), Old Grimsby Harbour (ogh), and West Broad Ledges (wbl).

Seagrass with abundant epiphyte cover was persistent at all sites throughout the length of the study. Time series of mean epiphyte cover, as well as mean seagrass density and the proportion of occupied patches at each site are shown in Fig. 2. In order to quantify empirical relationships in the data, we explored a mixed-effects time series model incorporating sampling year and sea surface temperature (SST) as explanatory variables, as well as autocorrelation (AR1) structure between years.

There was no overall, linear, temporal trend in epiphyte cover or any measure of seagrass abundance (Likelihood Ratio tests for slope parameter: epiphyte cover, L.R. = 1.95, *p* = 0.38; local seagrass density, L.R. = 3.58, *p* = 0.17; seagrass metapopulation occupancy, L.R. = 2.04, *p* = 0.36; ‘combined’ – see Methods – scale seagrass abundance, L.R. = 3.02, *p* = 0.22). However, we did find a small but statistically significant positive association between SST and local seagrass density (L.R. = 4.85, *p* = 0.028), although this was not evident for metapopulation occupancy (L.R. = 0.130, *p* = 0.72), combined scale abundance (L.R. = 0.737, *p* = 0.39), or epiphyte cover (L.R. <0.001, *p* = 0.98).

**Figure 3:**
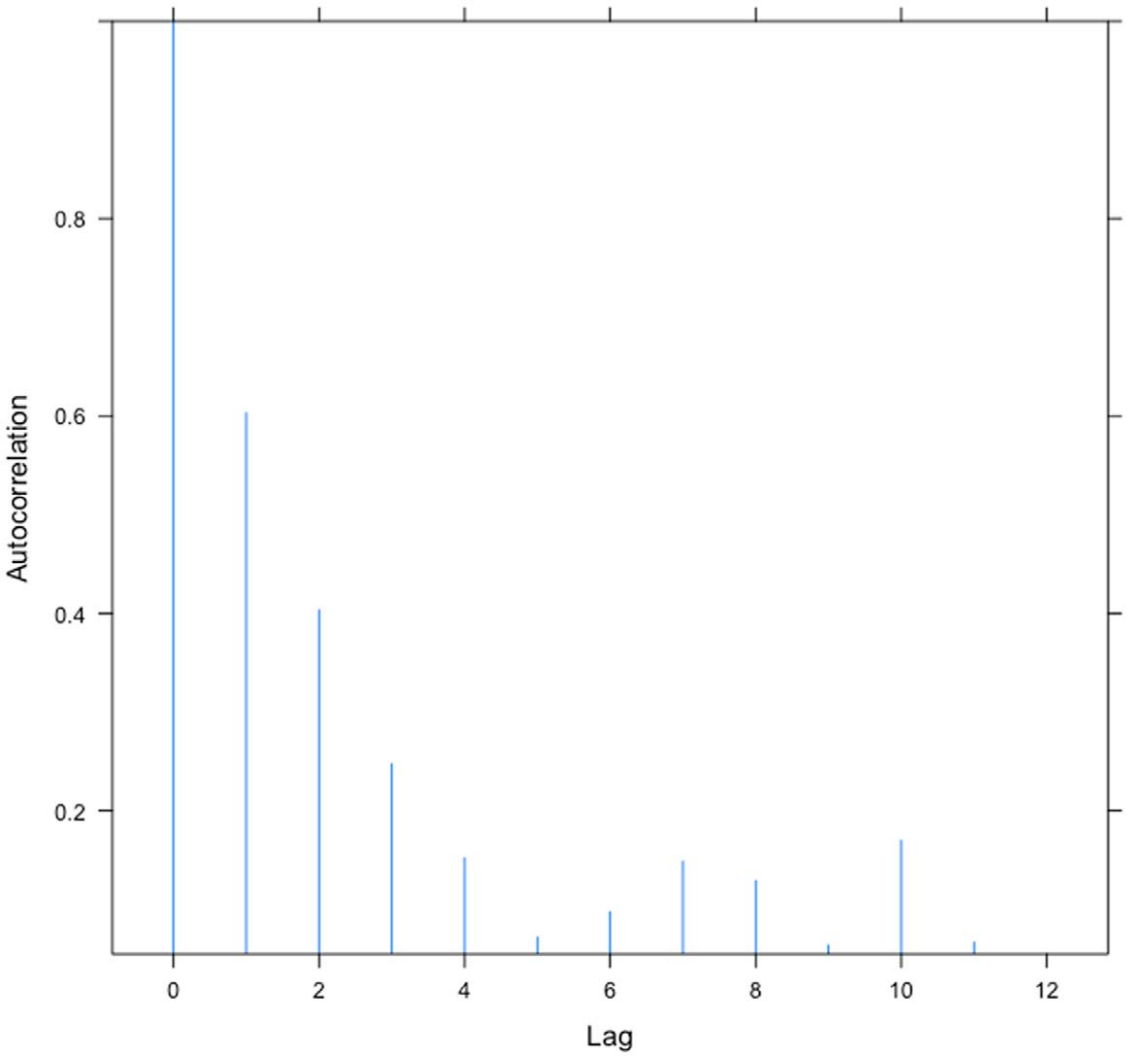
Seagrass metapopulation autocorrelations from the Isles of Scilly, UK. Average time-lagged empirical autocorrelations in the proportion of area (quadrats) occupied by seagrass (*Zostera marina*) within 16 year time series, sampled from five independent meadows around the Isles of Scilly.

We found no evidence of temporal autocorrelation within local seagrass density (ΔAIC = 0.39) or epiphyte cover (ΔAIC = 1.77). However, first order autocorrelation was strong within time series of metapopulation patch occupancy (ΔAIC = 21.0, Fig. 3), suggesting that patch turnover may operate over a slower time scale than dynamics driving local seagrass density. Not surprisingly, this pattern was also observed in time series of combined scale seagrass abundance, which incorporates the patch occupancy information (ΔAIC = 25.8).

These empirical differences in both fixed effects (year and SST) and temporal autocorrelation structure, evident between different measures of seagrass abundance, suggest multiple ecological processes operating at different spatial scales. We went on to explore the role of spatial scale using a simple population dynamic model fitted to our time series.

### Population Dynamics

We developed a Lotka-Volterra competition model in order to quantify intra- and interspecific competition between seagrass and its epiphytes over a range of spatial scales. The exponent of the intercept, *β*_0_, of our local density model indicates the net reproductive rate in the absence of density dependence. We estimated the net reproductive rate of *Z. marina* to be 3.06 (95% CI: 1.44, 7.27) year^−1^. We also quantified and compared factors that limit optimal growth at both the local and metapopulation scale.

The three measures of seagrass abundance, at different spatial scales, were correlated: Pearson’s *r*_(local density, metapopulation occupancy)_ = 0.33; *r*_(local density, combined abundance)_ = 0.59; *r*_(metapopulation occupancy, combined abundance)_ = 0.90. Therefore, we did not necessarily expect any measure of seagrass abundance to completely outweigh the other spatial scales when comparing (meta)population dynamic models. However, a difference in the relative weights of each measure of seagrass abundance is informative for determining the contributions of different spatial scales to dynamics [50]. Calculating Akaike Weights (AICcWt) between models incorporating each of the measures of seagrass abundance, we found that local seagrass density was clearly the dominant spatial scale limiting seagrass dynamics within occupied habitat patches (Table 1a), and similarly that metapopulation patch occupancy took the form of a stationary distribution with little influence from within patch seagrass density (Table 1b). Here, there was little evidence for any interaction between local and metapopulation scales. However, making the same comparison between seagrass abundance measures in the model explaining local epiphyte dynamics, we found close to equal weighting between all three spatial scales (Table 1c).

**Table 1:** Seagrass-epiphyte population dynamic modelling.

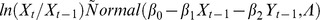 a) seagrass local population dynamics,				
spatial scale	AICc	ΔAICc	AICcWt	slope, *β*_1_ (SE)
local	27.54	–	0.99	0.320 (0.102)
metapopulation	38.81	11.27	0.00	0.111 (0.049)
combined	39.46	11.92	0.00	0.056 (0.038)
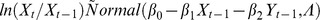 b) seagrass metapopulation dynamics,				
spatial scale	AICc	ΔAICc	AICcWt	slope, *β*_1_ (SE)
local	–70.32	–	0.90	0.056 (0.016)
metapopulation	–65.64	4.67	0.09	0.056 (0.022)
combined	–61.79	8.53	0.01	–0.082 (0.052)
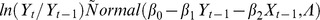 c) epiphyte population dynamics,				
spatial scale	AICc	ΔAICc	AICcWt	slope, *β*_1_ (SE)
local	199.64	–	0.34	0.445 (0.185)
metapopulation	199.72	0.09	0.33	0.880 (0.342)
combined	199.73	0.09	0.33	0.289 (0.158)

We explored three population dynamic models of intra- and inter-specific competition between seagrass and its epiphytes: (a) ln(*X_t_/X_t_*_-1_) represents the population growth rate of local seagrass density; (b) ln(*X_t_/X_t_*_-1_) describes the net colonization/extinction rate of available habitat by seagrass; and (c) ln(*Y_t_/Y_t_*_-1_) represents the population growth rate of epiphytes on seagrass leaves. In each case, we compared the relative weight of evidence (AICcWt) for local, metapopulation and ‘combined’ scale (average local density including unoccupied patches) seagrass abundance as drivers of population dynamics.

**Figure 4:**
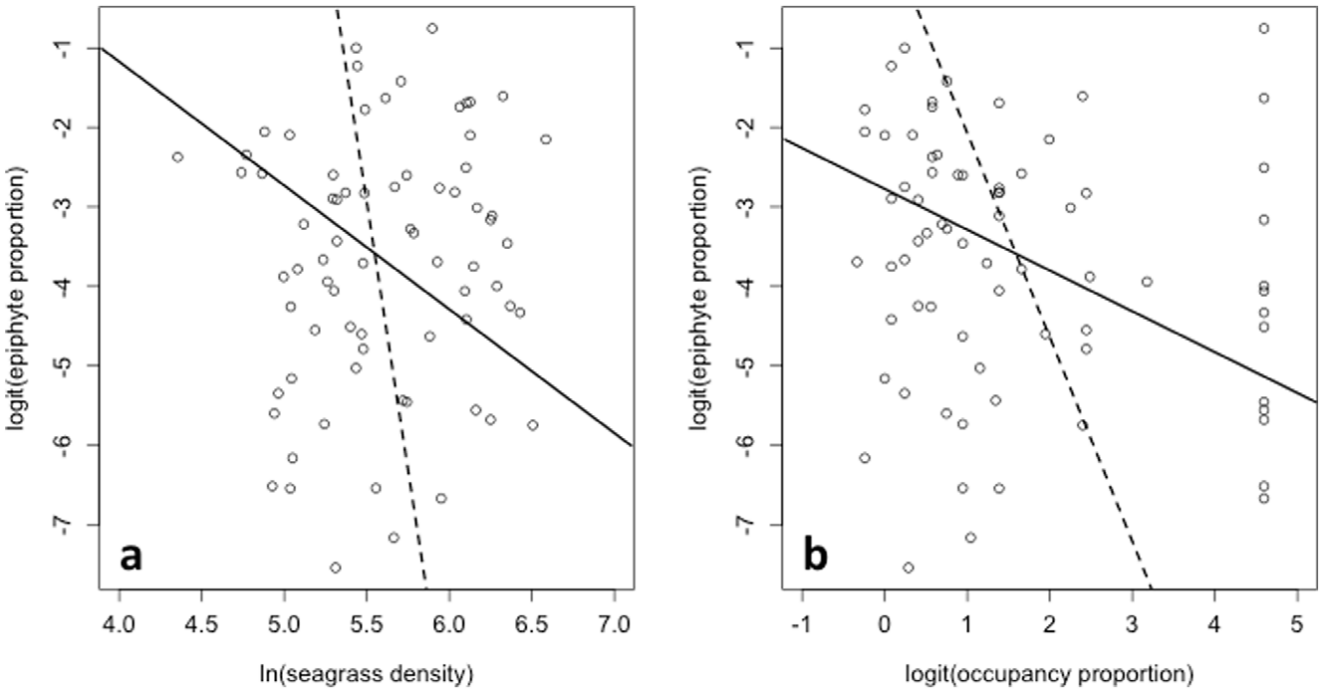
Phase plots of seagrass-epiphyte competition. Panel (a) average quadrat seagrass density plotted against epiphyte cover from 16 year time series, sampled at five independent *Zostera marina* meadows around the Isles of Scilly, UK; panel (b) the proportion of sampled area (quadrats) occupied by seagrass plotted against epiphyte cover (where seagrass patch occupancy proportion = 1, this was substituted with 0.99 for logit-transformation). Solid lines show epiphyte zero isoclines (no net population growth); dashed lines show *Z. marina* zero isoclines.

We went on to analyze seagrass and epiphyte inter-dependency using phase plots (Fig. 4). Here, zero-isoclines mark no net population growth of both seagrass and its epiphytes, parameterized from our fitted population model. The downward slopes of the epiphyte isoclines indicate that both local and metapopulation scale seagrass abundance limit epiphyte cover, with a slightly steeper slope attributed to local density. Likewise, non-vertical seagrass isoclines illustrate the negative effects of epiphyte cover on both local seagrass density and patch occupancy. On this axis, the scales, so slopes, are not comparable. We infer that competitive processes operating over multiple spatial scales affect epiphyte cover and this feeds back on both seagrass density and extent to drive long-term seagrass-epiphyte population dynamics.

## Discussion

In this study we investigated the long-term dynamics of a natural seagrass-epiphyte community. Seagrass and its epiphytes persisted in a competitive interaction, where stability was ensured by the domination of intraspecific (or perhaps more accurately in the case of epiphytes, intra-guild) competition within focal populations, compared to the effects of interspecific competition. In order to confirm that the Isles of Scilly *Zostera marina* population was comparable to healthy populations elsewhere, we estimated the maximum net reproductive rate of *Z. marina* in its natural setting. The estimated annual multiplication rate of ≈ 3 compares favourably with other measures of recruitment for this species (in the range 0–3), reviewed by Duarte et al. [51], and could be seen as a baseline figure from a ‘healthy’ population, against which studies of threatened populations might be compared. Our estimate averages over long-term stochastic fluctuations in a way that comparable measures from short-term studies do not. On the other hand, our model assumes no inter-annual survival of seagrass leaves (probably reasonable – Fig. 1 in [44] – but not confirmed) and does not partition between vegetative reproduction and recruitment from seeds. As with other aspects of our study, these findings provide a useful addition to information derived from detailed short-term investigations, rather than seeking to overturn them. Importantly, we also found that multiple spatial scales of seagrass distribution contributed to long-term epiphyte dynamics.

In contrast to studies of terrestrial systems, where the emphasis is on the effects of hosts on epiphytes, there is a history of investigating the contribution of epiphytes to overall seagrass ecosystem value; and of short-term, manipulative studies into the impact of epiphytes on seagrass growth. Epiphytes add primary productivity to the system [52]–[54], as well as providing a food source to a broader range of grazing invertebrate species than seagrass alone [55]. In addition, epiphytes contribute up to 60% of carbon flux in seagrass ecosystems [56]. The majority of studies (reviewed in [57]) suggest that where epiphytes influence seagrass growth and survival, the effect is negative. However, there is some evidence for the opposite effect: Fixation by epiphytes is thought to increase nitrogen availability to seagrass through the decomposition of dead epiphyte grazers [58]. In addition, data from Sand-Jensen [59] (Fig. 3 in that study) indicated that seagrass photosynthesis might actually proceed at a higher rate in plants with heavy epiphyte loadings than bare plants, although no mechanism for this was suggested. Conversely, seagrass may increase nutrient availability to their epiphytes by transporting nutrients from substrate to leaf surface [60] (somewhat analogous to the ‘habitat-tracking’ concept), as well as simply providing a stratum for epiphyte attachment (‘patch-tracking’).

These contrasting findings suggest that the relationships between seagrass and its epiphytes are highly non-linear over the full range on naturally occurring seagrass density. Our data are from a relatively pristine seagrass habitat and observed plant densities only span a fairly narrow range. Therefore we chose to model density dependence with linear functional forms but caution strongly against extrapolating this baseline model to seagrass that is heavily impacted by human interference or disease. Rather, our model forms an appropriate null hypothesis of the long-term interaction between seagrass and its epiphytes, against which studies of threatened or declining seagrass should be compared. In particular, many studies of seagrass-algae interactions are motivated by the widespread threat to seagrass by eutrophication. In such cases, excessive algal blooms threaten seagrass by reducing available light in the water column, smothering seagrass leaves and directly competing for nutrients [61].

In our system, while we found evidence of a reciprocal negative interaction between seagrass and its epiphyte community, at the same time both seagrass and epiphytes were persistent throughout the study period. Observed heterogeneity in equilibrium epiphyte abundance, both within and between meadows (Fig. 2), is likely to result from environmental factors such as turbidity. However, long-term seagrass-epiphyte coexistence is achieved through the balance of within and between species processes, here with intraspecific competition outweighing interspecific competition. This is illustrated using phase plots (Fig. 4), with zero isoclines – lines in parameter space denoting no net population growth – crossing at the predicted equilibrium abundances of seagrass and epiphytes. Hence while interspecific competition is evident in the long-term population dynamics, it has only a quantitative effect, modifying the equilibrium abundance, rather than a qualitative effect, leading to competitive exclusion. It would be interesting to investigate whether adverse environmental conditions, such as eutrophication, can push the relationship between seagrass and its epiphytes from one of stable coexistence towards competitive exclusion.

This long-term study was not aimed at uncovering specific mechanisms underpinning observed processes. However, there is a broad consensus that availability of light is the major limiting resource in healthy and undisturbed seagrass populations [51], [62]–[64]. Light attenuation is also likely to present a limitation to algal epiphyte growth, although it is largely unknown how much seagrasses compete with their epiphytes for nutrients [44]. Persistent coexistence in this seagrass-epiphyte system, regulated around a stable equilibrium, suggests that different resources ultimately limit seagrass and its epiphytes (strong interspecific competition for the same resource predicted to lead to competitive exclusion [65]). Consistent with this, epiphyte abundance is commonly related to nutrient levels in the water column [66] and even used as an indicator of eutrophication [67]. Competitive interference for nutrients between epiphyte species has been postulated by Romero et al. [68] but detailed knowledge of the factors that limit epiphyte growth is recognized as a knowledge gap.

In this study, we did not attempt to identify specific epiphytes, but rather treated all visible epiphytes as a functional group, likely to have a similar effect on seagrass growth by restricting light reaching the photosynthetic surface of leaves. In reality, the epiphytic community of *Zostera marina* is typical of many seagrasses, dominated by algae but comprising a range of invertebrate species as well [44]. There is known to be substantial spatial and temporal heterogeneity in epiphyte distributions on the leaves of *Z*. *marina* [69], [70]; a phenomenon also found in other seagrass genera, such as *Amphibolis* [71] and *Posidonia* [72]. This diversity in epiphytic species is likely to be structured by rich and, as yet, uncharted population dynamics. We have certainly observed between meadow variation in some species, particularly the snakelocks anemone (*Anemonia viridis*) and the nationally important stalked jellyfish species *Haliclystus sp.*, *Lucernariopsis campanulata* and *L. cruxmelitensis*, and recognize that further work is warranted to resolve fine grain epiphyte community structure. However, our finding that both local and metapopulation scale processes influence overall epiphyte abundance is consistent with the hypothesis that epiphyte dynamics are largely driven by recruitment [44], with metapopulation scale processes limiting epiphyte dispersal and local scale processes limiting vegetative reproduction in epiphytes.

Further, manipulative experiments would be needed to determine whether the influence of local vs. metapopulation scale processes is partitioned between different epiphytic species, or affect all epiphyte species similarly. The former would suggest that fluctuations in the total abundance of epiphytes should be accompanied by shifts in the composition and possibly species richness of the epiphyte community. However, in a nearby (Plymouth, UK), similar seagrass habitat, epiphyte composition was relatively uniform within seagrass meadows and 99% of *Z. marina* epiphytes were found to be filamentous or coralline algae [44]. More broadly, studies have found little variation in species composition at the local scale [45], [69], [73], [74], making differential responses between epiphyte species an unlikely explanation for the importance of multiple spatial scales observed in our study. This is supported indirectly by an earlier study of the infaunal (but not epiphytic) invertebrate community at three of our study sites (blt, htb, wbl), which found that between meadow differences substantially outweighed within meadow differences in assemblage composition, even though some within patch variation was observed [75]. Therefore, we infer from the relative invariance of the epiphytic community at the scale we measured it, that seagrass distribution (local density and regional distribution) is likely to affect both the dispersal and vegetative growth rates of individual epiphyte species, rather than for example local seagrass density limiting vegetative growth of one epiphyte group and seagrass patch distribution limiting dispersal success in a different group. However, this could only be confirmed by intensive surveys of epiphyte identities over a range of temporal and spatial scales.

Ultimately, epiphyte accumulation will be curtailed by longevity of seagrass. *Z. marina* is known to grow in annual or short-lived perennial forms, with individual leaves surviving for up to 100 days [44]. It is apparent from our study site that older seagrass leaves tend to support greater epiphyte cover and the shedding of these leaves provides a potential mechanism to mitigate the negative impact of epiphytes. Therefore, the survival of seagrass in a given situation is likely to have a substantial impact on epiphyte dynamics but this remains a knowledge gap in our system. More generally, epiphyte species richness has been shown to increase with time but no clear successional pattern is evident [76]–[78]. Our study was carried out at the same time each year, so largely controlling for within season accumulation (although there will inevitably be environmental stochasticity in this respect). While within season variation in epiphyte distributions is well charted [52], [53], [79]–[81] none of these provide the inter-annual data necessary to test hypotheses on long-term population dynamics.

In conclusion, our long-term study complements, and to an extent synthesizes, existing short-term investigations of specific mechanisms in the interaction between seagrass and its epiphyte community. We show how ecological processes, detailed elsewhere using manipulative studies, contribute to the inter-annual fluctuations of a natural ecosystem. This should help to establish baseline understanding of a globally important habitat and help to formulate predictions and responses to future threats and disturbances. Currently, there is an increased awareness of the need to understand the natural restoration potential of seagrass meadows [82] and our findings highlight the importance of measuring local density as well as wider extent in *Zostera marina* habitat in this context. It would also be useful to continue developing metrics to understand how ecological patterns and processes scale in seagrass meadows (for example, Cunha et al. [83], [84]). More broadly, particularly in marine environments, there is a shortage of data testing ecological theory on the roles of spatial scaling and structure, which is vital if conservation management is to be underpinned by relevant mechanistic understanding.

## Materials and Methods

### Seagrass Meadow Locations

We monitored five seagrass meadows around the Isles of Scilly, UK (Fig. 1), from 1996 to 2011, using consistent and rigorous survey methodology [33], [85]. Access to sampling sites is not a restricted activity and no permits were required. GPS positions and chart datum depths for the centres of these meadows are: Broad Ledges, Tresco (blt: 49° 56.4′ N, 06° 19.6′ W, depth: 0.2 m); Higher Town Bay, St. Martin’s (htb: 49° 57.2′ N, 06° 16.6′ W, depth: +0.5 m drying height); Little Arthur, Eastern Isles (la: 49° 56.9′ N, 06° 15.9′ W, depth: 1.0 m); Old Grimsby Harbour, Tresco (ogh: 49° 57.6′ N, 06° 19.8′ W, depth: 0.6 m); and West Broad Ledges, between Tresco and St. Martin’s (wbl: 49° 57.5′ N, 06° 18.4′ W, depth: 0.6 m). Mean low water springs is 0.7 m; mean high water springs is 5.7 m. Gradients across meadows are insubstantial, with typically less than 0.5 m depth variation across individual meadows.

### Survey Techniques

Seagrass (*Zostera marina*) was surveyed annually, during the first week of August, by placing 25 quadrats (0.0625 m^2^) in each meadow. Quadrat positions were predetermined as random rectangular coordinates (*x*, *y*) translated into polar coordinates (*distance*, *bearing*), radiating from the centre of the meadow. Randomization of quadrat locations was renewed each year and the maximum distance was 30 m from the focal point, close to the centre of each meadow.

Measurements recorded within each quadrat included: number of shoots (shoot density) visible above the surface of the sand (these are connected by unobserved networks of rhizomes in the substrate); the number of leaves per shoot; and length of the longest leaf on each shoot. ‘Shoot size’ was calculated as the number of leaves × longest leaf length on a given shoot and, hence, seagrass density as the sum of shoot sizes per quadrat.

Currently, there is substantial interest making global meta-analyses and developing universal metrics of seagrass of seagrass abundance [30], [57]. Consequently, for comparison with other studies, our ‘seagrass density’ was scaled to per square metre. *Zostera marina* leaves are strap like, showing no striking variation either along the length of the leaf or between leaves. An assumption of uniform leaf diameters would make this proportional to the Leaf Area Index found in studies of many other vegetation types. Finally, although we do not analyze it here, we also present time series of shoot density (shoots m^−2^) in addition to the fully worked-up ‘seagrass density’.

These seagrass meadows are typical of many marine landscapes [86], taking the form of patchily distributed vegetation, with bare sand forming the interstitial matrix [87]. Quadrats placed on sand were recorded as zero abundance. This allowed the relative contributions of local and metapopulation scales to be quantified. For each meadow, we calculated: the average seagrass density in occupied quadrats (local scale); the proportion of occupied quadrats in a meadow (metapopulation scale); and the average density in all quadrats, including empty quadrats (‘combined’ local and metapopulation scale).

In addition, we recorded the proportions of individual leaves visibly covered in epiphytic growth, based on an accepted categorization: (a = 0), (0<b<0.02), (0.02<c<0.25), (0.25<d<0.5), (0.5<e<0.75) and (0.75<f<1) [88]. Percentages were averaged by equating categorical scores to the mean of the corresponding proportion bracket.

### Population Modelling

We began with the familiar Lotka-Volterra competition model for two species, *X* and *Y*, competing for a shared, limiting resource [65]:



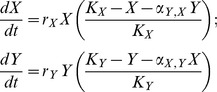
.

Here, *r* represents the exponential growth rate of the focal species in the absence of density dependence and *K* is the carrying capacity of the local environment that sets the equilibrium density in the absence of interspecific competition, *α*, from the other species. By integration [89], the discrete time analogue of this type of equation becomes:



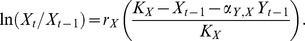
.

The discrete time equation can be fitted to time series data as a linear statistical model, taking the form:


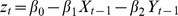
; where 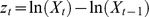, 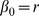, 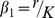, and 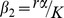.

At the metapopulation scale, the Levins model of patch occupancy can be written in an equivalent form [90]. In this case, *X* is the proportion of occupied patches. Where previously *r* = birth rate – death rate, here, *r* = habitat patch colonization rate – extinction rate, and *K* represents the equilibrium patch occupancy, usually assumed to result from density-dependent colonization.

Therefore, we developed three separate models: (a) seagrass within patch dynamics (*dX*/*dt* = rate of change of local density); (b) seagrass metapopulation dynamics (*dX*/*dt* = rate of change of the proportion of occupied patches); and (c) epiphyte dynamics (*dY*/*dt* = rate of change of epiphyte cover). In each case, our three different scale measures of seagrass abundance (‘local’, ‘metapopulation’, or ‘combined’) were incorporated separately as explanatory variables (nine models in total – see Table 1). We quantified evidence for the influence of each measurement scale using Akaike Weights for each set of three seagrass explanatory variables, based on the second order Akaike Information Criterion, AICc [50].

Linear models were fitted to our spatially replicated time series data in a mixed-effects framework [91]. Seagrass density as an explanatory (independent) variable was log-transformed; proportions of occupied patches and epiphyte cover as explanatory variables were logit-transformed. Spatial heterogeneity (within meadow heteroscedacity and between meadow correlation) was modelled with an empirical variance-covariance matrix, Λ. Gaussian noise has been shown to be appropriate descriptor of stochastic processes in spatially explicit systems [92].

All statistical and population dynamic modelling was performed using R 2.14.1 (http://www.r-project.org).
